# Microscopic Mechanism of Electrical Aging of PVDF Cable Insulation Material

**DOI:** 10.3390/polym15051286

**Published:** 2023-03-03

**Authors:** Zhiyi Pang, Yi Li, Hanbo Zheng, Rui Qin

**Affiliations:** 1 Faculty of Intelligent Manufacturing, Nanning University, Nanning 541699, China; 2 School of Mechanical and Electrical Engineering, Liuzhou Vocational & Technical College, Liuzhou 545000, China; 3 School of Electrical Engineering, Guangxi University, Nanning 530004, China; 4 Liuzhou Power Supply Bureau of Guangxi Power Grid Co., Ltd., Liuzhou 545000, China

**Keywords:** poly viny difluoride, electrical insulation, density functional theory, molecular simulation

## Abstract

In this study, the quantum chemical method was used to investigate the microscopic characteristics of α-poly viny difluoride (PVDF) molecules under the influence of an electric field, and the impact of mechanical stress and electric field polarization on the insulation performance of PVDF was analyzed through the material’s structural and space charge characteristics. The findings reveal that long-term polarization of an electric field leads to a gradual decline in stability and a reduction in the energy gap of the front orbital, resulting in the improved conductivity of PVDF molecules and a change in the reactive active site of the molecular chain. When the energy gap reaches a certain value, a chemical bond fracture occurs, with the C-H and C-F bonds at the ends of the backbone breaking first to form free radicals. This process is triggered by an electric field of 8.7414 × 10^9^ V/m, which leads to the emergence of a virtual frequency in the infrared spectrogram and the eventual breakdown of the insulation material. These results are of great significance in understanding the aging mechanism of electric branches in PVDF cable insulation and optimizing the modification of PVDF insulation materials.

## 1. Introduction

PVDF possesses unique piezoelectric, ferroelectric, and thermoelectric properties, as well as a low price, high flexibility, good biocompatibility, excellent aging resistance, and chemical stability [1,2]. Ferroelectric polymers such as PVDF are insulating, polar, and have a non-conjugated backbone from an electronic perspective [3], which makes them highly suitable for use as insulating materials [4,5]. The environment in which cables operate faces challenges such as climate extremes and corrosion [6,7], and insulation requires support from a wider range of properties, such as flame resistance and thermal stability. The PVDF material has multiple excellent properties, such as elasticity, a low weight, high chemical resistance, and heat resistance [8], so it is often used to produce cable insulation skins and printed circuit board insulation.

However, during the operation of DC cables in power transmission engineering, various factors, such as line faults and equipment switching, can cause the generation and invasion of an operation impulse voltage [9]. Moreover, the electromagnetic energy conversion process among the components causes the line system to oscillate, producing a high-frequency (hundreds to thousands of Hz) high-amplitude impulse voltage. Under the action of such repeated impact over-voltage, equipment insulation is damaged by long-term aging, and the cumulative effect of the impact voltage on equipment insulation damage requires urgent attention. The space charge effect is generally considered to be the main reason for the deterioration of insulation materials, especially DC insulation [10,11]. Discharge has a significant acceleration effect on the deterioration of polymer insulation. Therefore, under the action of electrical stress, PVDF often appears to age as an electrical insulation material, resulting in cracking and leading to premature insulation damage [12], which poses a serious threat to the safe and stable operation of electrical equipment and even the entire power system. The high temperature generated by the discharge causes the pyrolysis reaction of molecules, which leads to degradation [13]. The energetic particles generated by the discharge hit the macro-molecular chains, resulting in breakage. The corrosive substances produced by the discharge erode the polymer and eventually damage it. However, these theories are mainly based on experiments on the electric aging of insulating materials and the experimental measurement of related parameters, which cannot intuitively reveal the dynamic process and microscopic mechanisms of aging and cracking phenomena in polymers under the action of an electric field [14].

The cracking reaction of insulating polymers under the action of an electric field is a relatively complex physicochemical process. Traditional macroscopic experimental techniques have some limitations, so the microscopic dynamics of PVDF breakdown under an electric field cannot be observed intuitively. With the rapid development of computer technology and software engineering, many previous experiments can be further studied at the microscopic level. The theory of quantum mechanics means that molecular simulation technology is not only limited to the study of chemistry, but also extended to electrical, physical, biological and material science and other disciplines, promoting the development of interdisciplinary research and providing a new means for people to understand the material world, in addition to experimental methods and theoretical methods [15]. Many scholars have applied molecular simulation methods to the field of high-voltage insulation, such as the synergistic effect of electric fields and temperature on insulating oil [16,17], the microscopic mechanism of the overheating of insulating oil [18,19], the aging and degradation of insulating materials [20,21], and the diffusion of gas molecules in oil paper insulation systems [22,23]. Meanwhile, molecular simulation has been successfully used in the study of PVDF, the static and dynamical mechanical properties of PVDF [24], elastic properties of PVDF crystals [25], and the effect of the addition of ionic liquids to PVDF [26]. It is obviously feasible to apply the molecular simulation method to the study of the space charge formation and electric branch aging of PVDF cable materials. 

In this study, the molecular dynamics simulation method is used to construct a PVDF molecular model, and the semi-empirical method is used to optimize the structure of the model. Different levels of external electric fields are applied in the direction of the main chain of PVDF to study the changes in the total ground state stable energy, the dipole moment, the molecular polarizability, and the space charge of PVDF molecules.

## 2. Density Functional Theory and Basic Model Calculations

### 2.1. Density Functional Theory

Density functional theory is a widely used computational method for studying the electronic structures of multi-electron systems. It has found extensive application in the fields of physics and chemistry, particularly in the study of molecular and condensed matter properties. Density functional theory is widely used in condensed matter physics, computational materials science, and computational chemistry [27,28]. In this paper, the density functional theory method is used to calculate the electronic structures of molecules, in which the effect of the external electric field on the electronic structure can be added to the equation by the potential energy term. After applying the electric field, the Hamiltonian H of the PVDF molecular system is as shown in Equation (1).


(1) $$H=H_{0}+H_{int}$$

Here, *H*_0_ is the corresponding Hamiltonian when no electric field is applied, and *H*_*int*_ is the corresponding Hamiltonian when the external electric field interacts with the molecular system. Under the dipole approximation, the Hamiltonian corresponding to the interaction between the electric field intensity *F* and the PVDF molecular system can be expressed as in Equation (2).

(2) $$H_{int}=-\mu \times F$$

where *μ* is the molecular electric dipole moment and *F* is the electric field strength. The whole calculation process is completed using the Gaussian09W software package; the Multiwfn 3.7 software [29,30] and VMD 1.9.3 software [31] are used for further analysis.

### 2.2. Basic Model

**Figure 1:**
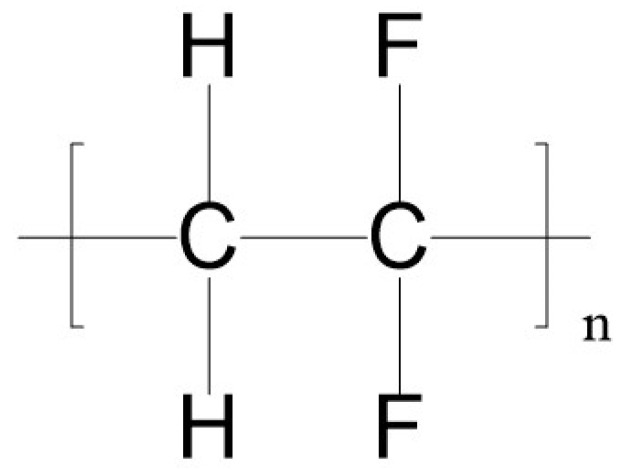
The repeating unit of PVDF.

**Figure 2:**
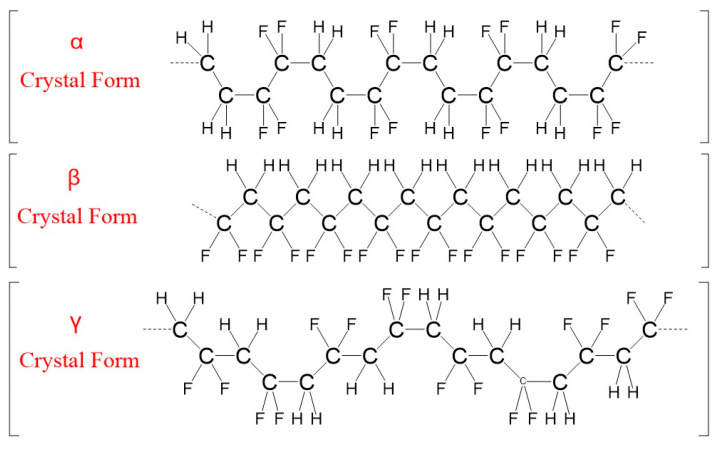
Molecular chain configuration of PVDF crystalline phase.

The repeating unit of the PVDF polymer is CH2-CF2, as shown in Figure 1. PVDF exhibits complex polymorphic characteristics, with five reported crystal structures, namely α, β, γ, δ, and ε [32]. The main crystal structures are α, β, and γ [33], as shown in Figure 2. The distinct crystal forms of PVDF display diverse properties, leading to varying physical and chemical properties and applications [34]. Therefore, it is crucial to investigate the impact of the different crystal structures on PVDF’s properties. The α phase is a thermodynamically stable crystal structure at room temperature and atmospheric pressure [35], and it is more stable than other phases in terms of thermodynamic properties; the most common form of PVDF crystal is the non-polar α phase, which can be used as an insulation material. However, it exhibits piezoelectric properties in the polar β phase and γ phase [36] and is commonly used as a piezoelectric material. In this study, a semi-empirical method is used to solve the simplified Schrodinger equation in order to describe the electron distribution, molecular structure, and properties. Under the premise of meeting the accuracy requirements, the PVDF molecules of the α-phase crystal form in Figure 2 are selected for in-depth computational analysis.

### 2.3. Molecular Model Computation

**Figure 3:**
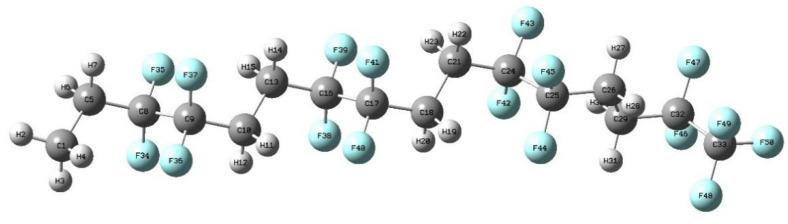
Molecular model of the α phase of PVDF.

**Figure 4:**
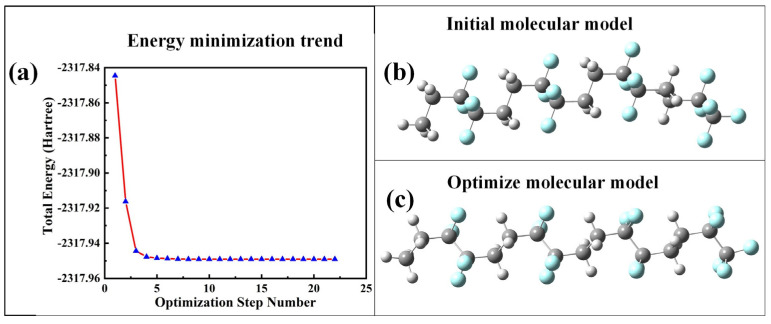
Optimization of the PVDF molecular model.

PVDF has a large molecular mass, and it is impractical as hundreds of thousands of molecules are required to perform simulations under different electric field strengths. In this study, models with different DPs were selected for pre-simulation. According to the results, it was found that the degree of polymerization had little influence on the results of the study. Combined with the previous analysis of the relationship between the aggregation degree and consumption of machine time, it is found that the value of the aggregation degree has little influence on the research content of this paper. Moreover, the purpose of this work is to study the overall effect of an electric field on the polymer, and an intramolecular study is not our primary purpose, so it is not necessary to use molecular models with tens of thousands of degrees of polymerization. Therefore, the α-phase molecular model of PVDF is constructed in this paper, as shown in Figure 3, to simulate the microscopic mechanism of PVDF molecules under the action of an electric field. Specific steps are as follows. The b3lyp/6-311g* method based on density functional theory is used to optimize the geometric configuration of the initial molecular model of PVDF (Figure 4b), and the stable conformation of molecules with the lowest energy is obtained. The molecular model is shown in Figure 4c, and the energy minimization trend is shown in Figure 4a, where gray represents carbon atoms, white represents hydrogen atoms, and blue represents fluorine atoms. The same method and base group are used to apply values from 0 to 0.0175 a.u. (1 a.u. = 5.142 × 10^11^ V/m) along the horizontal direction of the molecular chain, respectively. The optimization and single-point energy calculation are carried out, and the microscopic mechanism of the electrical aging of PVDF materials is studied. 

First, the changes in molecular structure are judged by the bond length, dihedral angle, and geometric structure. Then, the influence of the electric field on molecules is further determined by the molecular dipole moment, polarizability, molecular frontier orbital, and electrostatic potential surface. Finally, infrared spectra are used to verify the above conclusions.

## 3. Simulation Results and Discussion

### 3.1. Effect of Applied Electric Field on Molecular Structure

**Figure 5:**
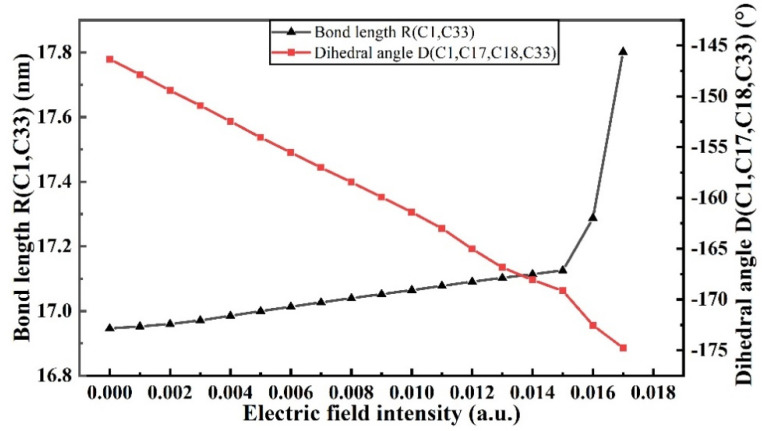
Changes in bond length and dihedral angle of PVDF molecules.

**Figure 6:**
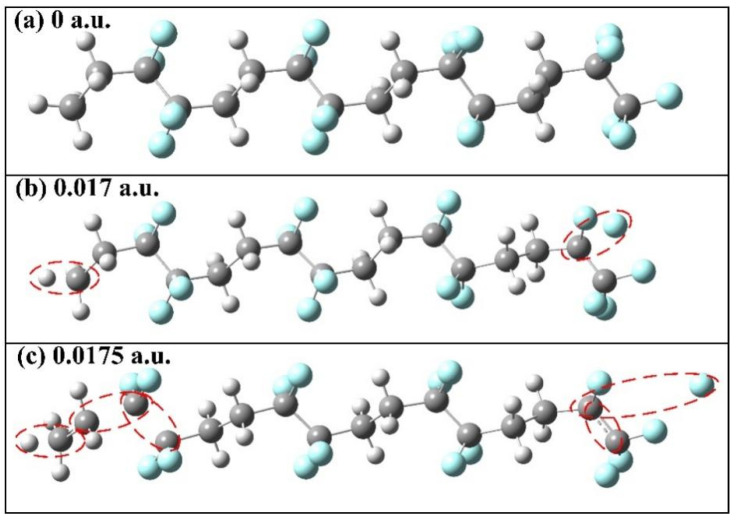
Molecular geometry at different electric field intensities.

Under the action of an external electric field, which is an electric dipole field, the chemical bond length and dihedral angle in the molecules will change accordingly, so as to characterize the change in the molecular geometric structure under the action of an electric field. *R*(C1,C33) represents the distance between the leftmost and rightmost C atoms of molecular chains, and *D*(C1,C17,C18,C33) represents the angle of twist of molecular chains. The specific changes in the bond lengths and dihedral angles of PVDF molecules are shown in Figure 5. The bond length of *R*(C1,C33) gradually increases with the increase in the intensity of the external field, particularly when the intensity of the external field increases from 0 to 8.7414 × 10^9^ V/m (0.017 a.u.), which is due to the transfer of positive and negative charges in the molecular system under the action of the electric field, producing the effect of a certain stretching on the molecular chain. *D*(C1,C17,C18,C33) changed from −146.341° to −174.769°, which gradually stretched the molecular structure and reduced the stability of the molecular geometric structure. The dashed circle represents that the chemical bond has been broken, when no external electric field is added, as in Figure 6a. When the external electric field is 8.7414 × 10^9^ V/m (0.017 a.u.), the C-H and C-F bonds at the left and right ends of the backbone are broken, as shown in Figure 6b. When the external electric field reaches 8.9985 × 10^9^ V/m (0.0175 a.u.), the internal molecular chain is broken, generating free radicals, as shown in Figure 6c, and the insulation is completely broken down.

### 3.2. Effect of External Electric Field on Total Molecular Energy, Dipole Moment, and Polarizability

**Figure 7:**
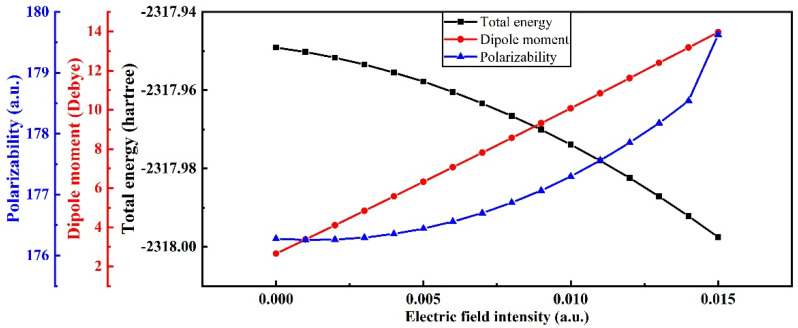
Variation in total molecular energy, dipole moment, and polarizability at different electric field intensities. (Polarizability: 1 a.u. = 1.6488^−41^ C^2^ m^2^ J^−1^).

The stability of the molecular system is related to the size of the total energy; the smaller the total energy is, the worse the stability of the molecular system is. Dipole moments and polarizability can characterize the spatial configuration and molecular polarity of molecules to some extent. Figure 7 shows the variation in the total energy, dipole moment, and polarizability of the PVDF molecular system with the electric field intensity. With the increase in electric field intensity, the total energy of the molecular system decreases gradually. This is because electrons are transferred along the electric field direction, making the charge on each atom in the electric field direction larger, increasing the dipole moment and polarizability of PVDF molecules. When the electric field intensity reaches above 8.2272 × 10^9^ V/m (0.016 a.u.), the molecular structure appears to have a virtual frequency, which means that the molecular structure has been damaged. Therefore, under the long-term action of the electric field, the stability of the PVDF molecular chain system will become worse. There is an upper limit for the molecular dipole moment. When the external electric field is too large, the dipole moment will break through the limit value, causing the electrons to eliminate the nucleus and form free electrons, which will eventually lead to the breakdown of the insulating medium.

### 3.3. Effect of External Electric Field on the Molecular Front Orbitals

**Figure 8:**
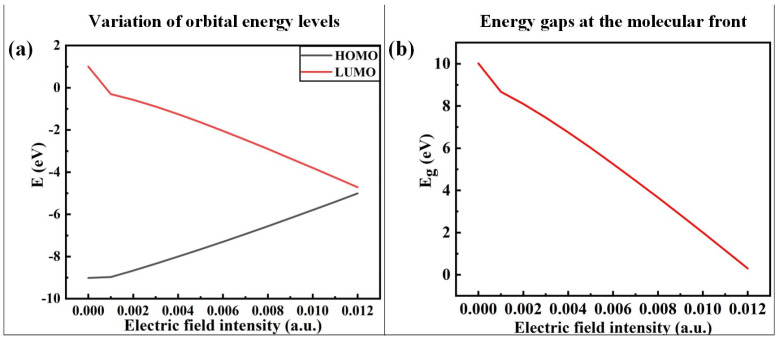
Variation in orbital energy levels and energy gaps at the molecular front at different electric field intensities.

In order to analyze the electronic motion characteristics of PVDF, the frontier orbital energy level, energy gap (Eg), and orbital composition of molecules under different external electric fields are calculated, respectively, and the calculation results of the energy level and Eg are shown in Figure 8. The size of Eg is defined as the difference between the energy levels of the lowest unoccupied orbital (LUMO) and the highest occupied orbital (HOMO). According to the frontier orbital theory [37], the higher the energy of an electron in the HOMO orbital of molecules, the less bound it is, and the easier it is for the electron transition to occur. The lower the energy of the LUMO orbital, the easier it is to accept electrons. Figure 8a shows that the HOMO energy level of PVDF increases with the increase in the electric field strength, indicating that the electrons of its orbital are more prone to transition. The LUMO energy level decreases continuously with the increase in electric field strength, indicating that its orbital is easier to obtain electrons. The Eg reflects the ability of electrons to transfer from occupied orbitals to empty ones. The smaller the Eg, the more easily the electron is excited and the more reactive the molecule is. It can be seen from Figure 8b that the Eg of PVDF gradually decreases with the increase in electric field intensity, indicating that the activity of the chemical reaction of molecules is continuously enhanced and the stability is reduced. When the electric field exceeds 6.1704 × 10^9^ V/m (0.012 a.u.) in the simulation calculation, the optimization does not converge, indicating that the structure is on the verge of failure.

**Figure 9:**
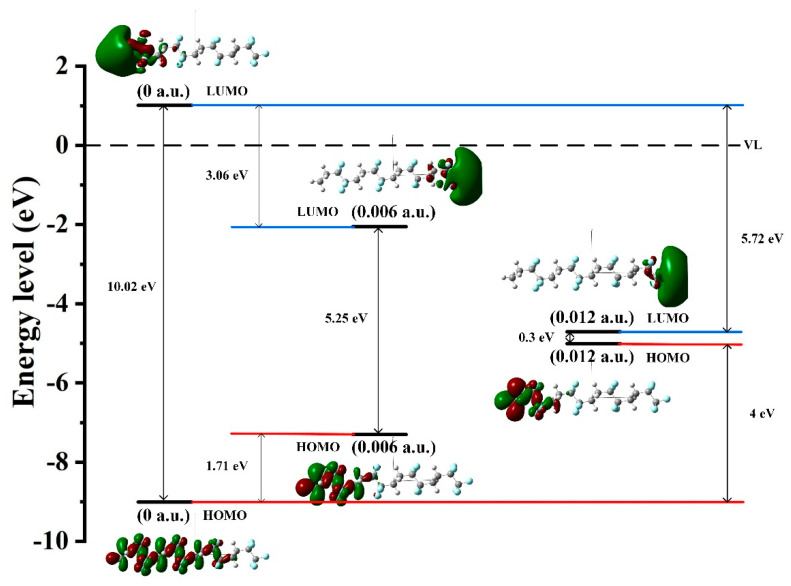
The energy level distribution and the frontier orbital of PVDF.

**Figure 10:**
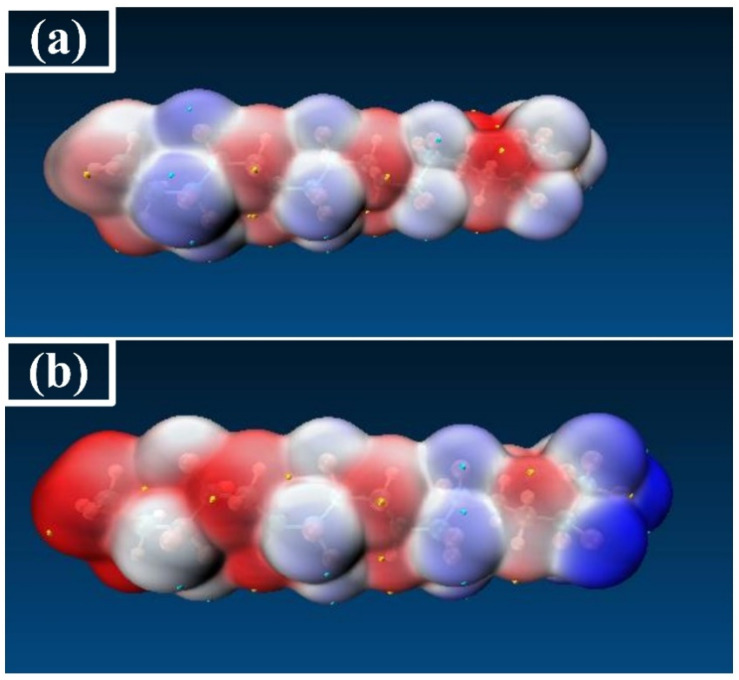
The electrostatic potential surface of PVDF.

In order to analyze the microscopic change characteristics of the space charge inside PVDF under a continuous electric field, this paper uses the changes in the trap energy level, orbital cloud map, and electrostatic potential surface as a basis for discussion. Figure 9 shows the energy level distribution and frontier orbit map (MO) distribution of PVDF when the electric field intensity is 0, 3.0852 × 10^9^ V/m (0.006 a.u.), and 6.1704 × 10^9^ V/m (0.012 a.u.), respectively, which can more intuitively reflect the motion characteristics of the space charge at the microscopic level. It can be seen from the distribution of MO map that the HOMO orbital and the LUMO orbital are mainly concentrated on the left end of the molecular chain before the electric field is applied, which is easily attacked by electrophile reagents and electrophilic reactions occur. However, the effect of the external electric field causes the distribution of the molecular front track to change greatly, and the active reaction site of molecules also changes accordingly, as shown in Figure 9. When the electric field strength is 3.0852 × 10^9^ V/m (0.006 a.u.), the LUMO orbital shifts in the opposite direction. When the electric field reaches 6.1704 × 10^9^ V/m (0.012 a.u.), the LUMO orbital moves towards the end of the chain, and the HOMO orbital also moves to the left end of the chain. The left and right sides of the molecular chain show nucleophilic and electrophilic activity, respectively. Without an external electric field, the electrostatic potential distribution on the molecular surface is as shown in Figure 10a. When the electric field reaches 6.1704 × 10^9^ V/m (0.012 a.u.), the molecular chain shows a more positive electrostatic potential at the left end and a more negative electrostatic potential at the right end, as shown in Figure 10b, which also corresponds to the performance of the molecular trap energy level mentioned above. This phenomenon indicates that the applied electric field leads to the continuous decrease in the LUMO energy level and the increase in the HOMO energy level, which will also change the active site of the PVDF reaction. When the electric field reaches 6.1704 × 10^9^ V/m (0.012 a.u.), the change in energy level distribution is more obvious. At the same time, the HOMO and LUMO orbital energy levels are very close, and the Eg between the HOMO orbital and LUMO orbital is only 0.95eV. At this time, PVDF will be in the semiconductor state. Electrons can move freely in the valence band and the conduction band to form a current, and the insulation performance of PVDF will fail.

To further predict the active reaction sites of molecules, the Hirshfeld method in Multiwfn 3.7 is used for electric field intensities of 0 and 6.1704 × 10^9^ V/m (0.012 a.u.). The contribution of each atomic orbital in the frontier orbital composition of PVDF is analyzed, and the results are shown in Table 1. According to the previous analysis, under the action of an external electric field, the HOMO and LUMO orbitals of molecules move to both ends of the molecular chain, respectively. When the electric field strength is 6.1704 × 10^9^ V/m (0.012 a.u.), the HOMO and LUMO orbitals are essentially concentrated on both ends of the molecules, enhancing the reaction activity of the molecular chain end. It can be found from Table 1 that the HOMO orbital of PVDF is mainly contributed by C and H atoms on the left side of the molecular chain, among which the contribution of C1, C5, C8, and C9 atoms is 51.15%, and the contribution of H4, H2, and H6 atoms is 33.16%. This indicates that C1, C5, C8, and C9 and H4, H2, and H6 atoms have strong reactivity on the left side of the PVDF molecular chain. Similarly, the main contribution of the LUMO orbital comes from the C atom and F atom on the right side of the molecular chain, of which the contribution rate of C33 and F50 atoms reaches 33.89% and 38.89%, respectively. At this time, the C-H bond on the left side and the C-F bond on the right side of the PVDF molecular chain are on the verge of fracture. This corresponds to the case of the breaking of the chemical bonds of the molecules, shown in Figure 6b.

**Figure 11:**
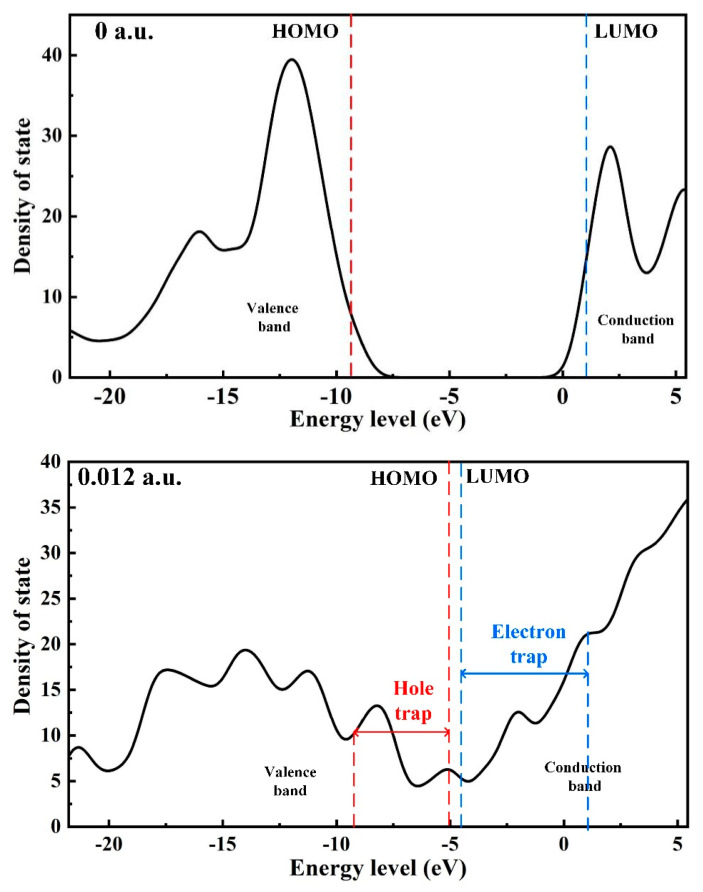
The density of states at 0 a.u. and 0.012 a.u.

The density of states (DOS) mainly refers to the density of the distribution of molecular orbitals at different energy levels. The density of molecular states can intuitively reflect the distribution of hole traps and the electron trap energy level density of molecular orbitals, and each peak can represent the distribution of a trap energy level [38]. The HOMO and LUMO orbital energy levels correspond to the positions of the valence band top and conduction band bottom, respectively. Under the action of an electric field, a certain amount of deep traps and distribution of the shallow trap energy level appear near the valence band and the conduction band, as shown in Figure 11. With the continuous action of the electric field, the density of trap energy levels formed by molecular orbitals will further increase. The HOMO orbital energy level shifts to a higher energy level, which introduces more hole traps near the valence band. The LUMO orbital energy level moves to the direction of the lower energy level, and more electron traps are introduced near the conduction band. Finally, the number of electron traps is higher than the number of hole traps. This difference between the number of holes and electron traps can indicate that the effect of the electric field will strengthen the ability for electronic transition inside PVDF, and it is easier to capture the free electrons or injected charges in the insulation material.

### 3.4. Effect of External Electric Field on Infrared Spectra 

**Figure 12:**
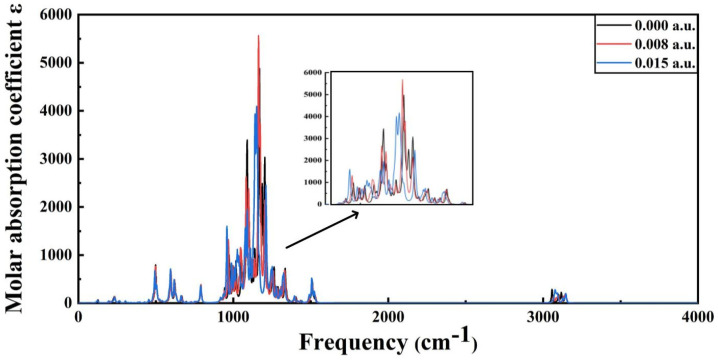
The infrared spectra of PVDF at 0 a.u. to 0.015 a.u.

**Figure 13:**
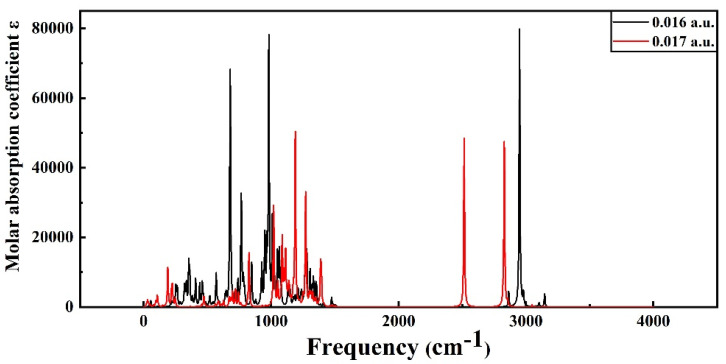
The infrared spectra of PVDF at 0.016 a.u. and 0.017 a.u.

The infrared spectra of PVDF under different electric field intensities are shown in Figure 12 and Figure 13. The infrared spectra of molecules under a low electric field intensity and high electric field intensity are compared with the infrared spectra under the condition of no electric field as the reference. As can be seen from Figure 12, when the electric field intensity is below 7.713 × 10^9^ V/m (0.015 a.u.), the infrared spectrum does not change greatly, indicating that the intensity of the low electric field has little influence on the molecular structure, and the molecular system still maintains the stability of the structure. However, when the electric field intensity is above 8.2272 × 10^9^ V/m (0.016 a.u.), the infrared spectrogram changes greatly, as shown in Figure 13. The main difference is that the peak value of absorption fluctuates greatly, the infrared activity of the vibration mode in many intervals is significantly enhanced, and the peak value of the corresponding absorption peak is significantly increased. The stretching vibration is intensified at C-H at 2949 cm^−1^ and C-F at 678 cm^−1^ at the end. Virtual frequency does not represent any real vibration; when the structure of the molecules is no longer stable, virtual frequency will appear in the infrared spectrum. When the electric field intensity continues to increase, the virtual frequency appears in the infrared spectrum, indicating that the structure of molecules is not stable and structural damage has occurred. The critical external electric field of the molecular space’s structural destruction is the starting point of insulation material aging, which will inevitably lead to the destruction of cable dielectric materials, thereby reducing the electrical strength of cables and finally causing the polymer insulation breakdown phenomenon.

## 4. Conclusions

The density functional method was utilized to investigate the microscopic characteristics of molecules inside the dielectric system of a PVDF cable under the influence of an external electric field. The effects of electric field intensity on the molecular structure, the total energy of PVDF materials, the polarization phenomena, the energy gap, and the infrared spectra were examined. The formation mechanism of electric branches within the cable’s dielectric system was analyzed at the microscopic level, leading to the following conclusions.

(1) It can be seen from the change in the orbital energy of the molecular front that with the increase in the electric field, the activity is enhanced, and the physicochemical reaction is more likely to occur, which destroys the optimal, stable structure of molecules, so the corresponding infrared spectra will also change.

(2) Under the influence of the external electric field, the dipoles of PVDF molecules rotated in a directional manner, generating an equivalent polarized space charge inside the cable’s dielectric system. A higher polarization rate and polarization space charge density were observed with an increased electric field intensity, which had an impact on the dielectric insulation performance.

(3) Strong electric fields were found to cause the cleavage and breakage of PVDF molecular chains, with the C-H and C-F bonds at the ends of the molecular chain being the first to break. From the front track and electrostatic potential surface, it was inferred that these chemical bonds had strong chemical reactivity and were more susceptible to reaction. Future modification technology may help to prevent breakdown at these weak points [39]. These findings can provide theoretical support for relevant practical engineering tests and targeted testing conditions, rather than blindly imposing external test conditions.

## Figures and Tables

**Table 1:** Front track composition.

0 V/m				6.1704 × 10^9^ V/m			
HOMO/%		LUMO/%		HOMO/%		LUMO/%	
C9	9.12%	H2	14.24%	C1	26.22%	F50	38.39%
C8	8.97%	H6	10.48%	H4	14.03%	C33	33.89%
C10	6.26%	C5	10.05%	C5	13.34%	F49	6.70%
F35	6.12%	C8	9.15%	H2	9.96%	F48	6.50%
C5	5.87%	H7	6.43%	H6	9.17%	C32	4.82%
F36	5.66%	C9	6.39%	C8	7.22%	F46	4.04%
C16	5.39%	H3	5.53%	C9	4.37%	F47	3.41%
